# Factors influencing childhood vaccine hesitancy and interventions for parents and caregivers from ethnic minority backgrounds in the United Kingdom to encourage uptake: a systematic review

**DOI:** 10.3389/fpubh.2026.1929472

**Published:** 2026-10-12

**Authors:** Tarela Juliet Ike, Dung Ezekiel Jidong, Callistar Kidochukwu Obi, Chiyem Lucky Nwanzu, Akaba Kingsley, Mulugeta Tamire, Marekegn Habtamu, Mieyebi Lawrence Ike, Evangelyn Ebi Ayobi, Peremi Richmond Ike, Rukevwe Francis Doghor, Rakhshi Memon, Kelvin Odeyovwi Ayobi, Aboneh Solomon Sime, Otovwe Agofure, Jo Parsons, Sarah Edwards, Maureen Iru Ntaji, Nusrat Husain

**Affiliations:** 1 Division of Psychology and Mental Health, University of Manchester, Manchester, United Kingdom; 2 School of Social Sciences, Humanities and Law, Teesside University, Middlesbrough, United Kingdom; 3 Division of Psychology and Mental Health, University of Manchester, Manchester, United Kingdom; 4 Department of Economics, Faculty of the Social Sciences, Delta State University, Abraka, Nigeria; 5 Department of Psychology, Delta State University, Abraka, Nigeria; 6 Department of Haematology/ Chief Medical Centre, University of Calabar, Calabar, Nigeria; 7 Federal Medical Centre, Ovwian, Nigeria; 8 School of Public Health, Addis Ababa University, Addis Ababa, Ethiopia; 9 The Leprosy Mission International, Ethiopia, Ethiopia; 10 Western Governors University, Salt Lake City, UT, United States; 11 Manchester Global Foundation, London, United Kingdom; 12 Tare Wyd Legal Chambers, Warri, Nigeria; 13 Beryl Foundation, London, United Kingdom; 14 University College London, London, United Kingdom; 15 Department of Public Health, University of Delta, Agbor, Delta, Nigeria; 16 School of Social Policy, University of Birmingham, Birmingham, United Kingdom; 17 Department of Community Medicine, Delta State University Teaching Hospital, Oghara, Delta, Nigeria; 18 Division of Psychology and Mental Health, University of Manchester, Manchester, United Kingdom; 19 Mersey Care NHS Foundation Trust, Liverpool, United Kingdom

**Keywords:** childhood vaccine hesitancy, ethnic minority, intervention, United Kingdom, vaccine uptake

## Abstract

**Background:**

Childhood vaccine hesitancy remains a significant public health concern, especially as it relates to ethnic minority population in the United Kingdom, who are reluctant to engage in uptake and record a higher number of unvaccinated children. Yet, there remains a significant research gap on the factors driving ethnic minorities parental hesitancy of childhood vaccination and interventions to improve uptake for the affected population in the UK, which is the primary aim and contribution of the present review.

**Method:**

The review strategy was underpinned by the Population, phenomenon of Interest and Context (PICo) model. Hence, the Population (parents and caregivers from ethnic minority backgrounds), the phenomenon of Interest (factors fostering parental childhood vaccine hesitancy and interventions to encourage uptake), and the Context (Vaccine hesitancy). For qualitative studies, the PICo strategy, which is a modified version of PICO, was used. Boolean operators (AND/OR/NOT) were used to ensure rigor and were adopted in the formulation of search terms. Eight databases were searched (PubMed, CINAHL, the Cochrane Controlled Trial Register, Embase, Web of Science, PsycINFO, ProQuest Central, and Scopus) for published articles between 2002 and June 2026. *N* = 10 studies met the inclusion criteria, and the relevant data were synthesized using a thematic synthesis approach. The review protocol was pre-registered on the PROSPERO Registry (no. CRD420261358747).

**Findings:**

The synthesized studies and analyses yielded the following main findings. (i) Skepticism about vaccines, (ii) Distrust in health professionals and policy, (iii) Poor understanding and limited information about vaccines, (iv) Social network and family influence and (v) communication-based intervention.

**Conclusion:**

Childhood vaccine hesitancy remains a challenge; the review recommends culturally adapted public health promotion messages and interventions to encourage uptake, to be tested using randomized controlled trials.

## Introduction

Parental vaccine hesitancy is an issue of significant global concern. Recent data from the United Kingdom Health Security Agency (1) suggest that 1 in 5 children start primary school not fully protected, and none of the childhood programs meets the 95% herd immunity target recommended by the World Health Organization. Of central concern is childhood vaccine uptake among ethnic minority populations in the UK, which has consistently been lower than among White British groups, with significant disparities observed across routine immunizations. In a study by Zhang et al. (2), drawing on data from 1,170,804 children born after 1 April 2006, children of mothers in 9 minority ethnic groups had lower vaccination coverage (61.3–97.5%) than the White British group (79.9–97.8%) for all vaccines. Bangladeshi, Indian, Chinese, Pakistani, and other Asian backgrounds, and White and Asian ethnic groups had similar vaccination coverage to the White British group (above 90% for most vaccines). However, significant inequities particularly affected the Caribbean group (e.g., 61% coverage of the 6/5/4-in-1 full course by children’s fifth birthday; RR 0.66, 95% CI 0.6–0.74 compared with their White British counterparts) (2). Such inequities extend to any other Black, African and Caribbean background who are reported to have a coverage of 68% for the MMR primary course (2). Of central concern are the factors driving hesitancy and interventions to improve uptake among ethnic minority populations in the UK, which this systematic review specifically seeks to address.

A growing body of literature has sought to explore the factors behind the disparities in childhood immunizations and highlight how ethnicity-specific factors such as migration history (3), religion (4), and language barriers (5) influence parents’ perceptions of vaccine acceptability, understanding of immunization schedules, and concerns about biological differences, ultimately contributing to lower uptake among Black and Asian families. Several studies have also highlighted the role of a wide range of structural and social barriers that intersect to shape these trends. These include perceived mistrust in government institutions (6), pharmaceutical companies, and healthcare systems (7)—rooted in historical and contemporary experiences of systemic racism (37). These factors arguably remain among the drivers of hesitancy among minority communities.

Another strand of literature stresses additional barriers, including low confidence in vaccines and heightened concerns about risks and side effects (6, 8). Access-related challenges such as inconvenient clinic hours, difficulties navigating NHS processes (9), and limited communication from trusted providers (6, 10) further compound hesitancy, particularly among newly arrived migrants, socioeconomically disadvantaged families, and groups already uncertain about vaccination. This is compounded by a perceived lack of culturally tailored, multilingual, and non-stigmatizing communication, which reflects poor public health messaging (11). This appears to be a key obstacle to effective engagement, highlighting the need for trusted community leaders, GPs from within target groups, and faith-aligned messaging to build confidence and uptake. Despite these challenges, evaluations of existing community-based interventions to address vaccine hesitancy among this population and existing interventions (e.g., community-based interventions) to encourage uptake remain lacking.

Against the backdrop stated above, this systematic review makes an original contribution by synthesizing the barriers to childhood vaccine uptake, including parental hesitancy, and the interventions to address them. This systematic review is also significant in its primary focus on synthesizing barriers and interventions targeting parental childhood vaccine hesitancy within ethnic minority populations in the United Kingdom, a group that remains disproportionately affected by lower vaccination uptake and poorer health outcomes. Unlike prior reviews that predominantly focus on addressing COVID-19 Vaccine hesitancy (12) or general population barriers to uptake of childhood vaccines globally (13), this review centers the socio-cultural, behavioral, and structural determinants shaping vaccine decision-making, thereby advancing a more nuanced, equity-oriented understanding grounded in lived experience and context. By integrating insights from diverse psychosocial strategies, such as community engagement, culturally tailored communication, trust-building initiatives, and behavioral change frameworks, the review builds an evidence base to inform the design of inclusive, culturally responsive interventions. Its significance also lies in bridging a critical gap between policy, practice, and marginalized communities, offering actionable knowledge to reduce health disparities, improve vaccine confidence, and ultimately prevent avoidable morbidity among underserved populations in the UK. In essence, the primary research question this systematic review seeks to address is:

What are the factors and barriers influencing parents from ethnic minority backgrounds’ hesitancy towards childhood vaccination, and what are interventions to encourage uptake in the United Kingdom?

For this review, we adopt the term ethnic minority as defined by the UK Race Disparity Unit (14), including Black people, Asian people, and people who do not identify as White. The review commences with a report on the methodology. It then presents the findings and discussion. The review concludes with policy recommendations, including those for service providers providing services for the affected population.

## Method

### Protocol/search strategy

The review protocol was developed in accordance with the Preferred Reporting Items for Systematic Reviews and Meta-Analyses (PRISMA) guidelines, which provide a robust, internationally recognized framework to enhance methodological rigor, transparency, and reproducibility in systematic reviews. By adhering to PRISMA, the study ensures that each stage of the review process—from study identification and screening to eligibility assessment and inclusion—is clearly documented and replicable by other researchers, thereby strengthening the credibility of the findings (38). In line with best practice, we established a comprehensive protocol before commencing the review to define the inclusion and exclusion criteria, outline the search strategy, and specify the analytical approach, minimizing bias and promoting consistency throughout the research process (44). Importantly, this protocol was formally pre-registered with the International Prospective Register of Systematic Reviews (PROSPERO; registration number CRD420261358747), reinforcing the study’s commitment to transparency, preventing selective reporting, and enabling replicability of the review methodology.

### Study’s inclusion and exclusion criteria

The review’s main aim was to examine the factors and barriers influencing ethnic minorities parents’ hesitancy towards childhood vaccination and the interventions to encourage uptake in the United Kingdom. Therefore, the search terms were designed to ensure an in-depth body of literature relevant to the review. Hence, the inclusion criteria were: (i) Qualitative, Mixed methods, or Quantitative studies of any design focusing on barriers to vaccine uptake or interventions to address childhood vaccine hesitancy for ethnic minority populations in the United Kingdom. This includes cohort, observational, cross-sectional, longitudinal, and population survey studies. (ii) Publications that are available in full text, are peer-reviewed or academic articles, (iv) Studies that include participants from the ethnic minority population whose children are partially or not up to date with vaccines and affected by vaccine hesitancy. (v) Studies conducted in the United Kingdom and (vi) Studies that include any form of community-based interventions, behavioral interventions, public health messaging interventions, tele-messaging, educational interventions, media campaigns, or psychosocial interventions (e.g., cognitive behavior therapy, community advocacy, psychoeducation) and sample from the affected populations in the United Kingdom.

The exclusion criteria of the review include: (i) Studies that are not focused on barriers influencing childhood vaccine uptake or interventions to address childhood vaccine hesitancy for ethnic minority populations regardless of gender in the United Kingdom and where the population is not focused on ethnic minorities as it relates to vaccine hesitancy; (ii) Unpublished studies (e.g., poster abstracts, blogs, conference abstracts); (iii) Studies not published in English and with no translation version; and (ii) Studies not meeting the review inclusion criteria.

The review focuses on synthesizing studies published between 2002 and June 2026, a timeframe deliberately chosen to include contemporary, policy-relevant evidence while keeping the literature manageable and methodologically coherent for analysis.

### Search strategy

The review adopted the PICo model (Population: parents and caregivers from ethnic minority backgrounds; phenomenon of Interest: factors fostering parental childhood vaccine hesitancy and interventions to encourage uptake; Context: childhood vaccine hesitancy) (39). The Boolean operators (AND/OR/NOT) were also adopted to design strategic search terms (40). Below are examples of the search strategies adopted for the review (Table 1).

**Table 1:** Showing sample search term adopted.

**Data base**	**Search terms**	**Date searched**	**Result returned**
**PubMed**	(“Parents”[MeSH] OR “father”[tiab] OR “mothers”[MeSH] OR “caregiver”[tiab] OR “childhood vaccine resistance”[MeSH] OR “childhood vaccine hesistancy”[tiab] OR “vaccine resistance”[MeSH] OR “vaccine hesistancy”[tiab] OR “England”[tiab] OR “Wales”[tiab]) (“Mothers”[MeSH] AND “Carer” [tiab] OR “Caregiver” [MeSH] OR “childhood vaccine hesitancy”[tiab] OR “childhood vaccine scepticism”[MeSH] OR “United Kingdom”[tiab]) (“public health Intervention”[MeSH] AND “vaccine hesitancy”[tiab] AND “Wales”[MeSH], AND “England” [tiab] AND “Northern Ireland” [tiab] AND “Scotland” [tiab] OR “parents” AND “child” [tiab] AND “childhood vaccine hesitancy” [tiab] AND “vaccine uptake” [tiab])	04/0/2026	33,487 6,011 1
**CINAHL**	“Vaccine hesitancy intervention” AND “England” NOT “Canada” OR “Australia” OR “Asia” OR “USA” AND “nonclinical intervention for vaccine hesitancy” OR “educational intervention” “communication campaign intervention”	05/04/2026	9
**Cochrane Controlled Trial Register**	“Media campaign intervention” OR “community-based intervention” OR “art-based intervention” AND “parent” AND “Vaccine hesitancy” “Caregivers” OR “childhood vaccine hesitancy intervention” OR “public health intervention” AND “Wales” OR “United Kingdom”	04/06/2026	9687 (trials) 100 (Cochrane reviews) 25,678 (trials) 250 (Cochrane reviews)

The search strategy was systematically developed using carefully defined search terms, which were applied across a comprehensive range of major electronic databases, including PubMed, CINAHL, the Cochrane Controlled Trials Register, Embase, Web of Science, PsycINFO, ProQuest Central, and Scopus, to ensure broad coverage of relevant literature. To further enhance the completeness and robustness of the search process, we supplemented database searching with manual screening of the reference lists of included studies, including Google Scholar, to identify additional relevant articles that may not have been captured through electronic searches alone. In addition, we made targeted visits to local academic resources, including the Teesside University Library in Middlesbrough, to access any other potentially relevant materials not readily available online.

### Included studies screening and selection

In total, 78,223 records were identified through the database and supplementary searches, yielding 19,307 studies. After removing duplicates and screening out studies that did not meet the inclusion criteria (n = 59), the remaining records underwent rigorous screening. Two independent reviewers systematically examined the titles and abstracts of all studies against the predefined inclusion and exclusion criteria to ensure consistency and prevent selection bias. We excluded studies that lacked empirical data or did not meet the eligibility criteria at this stage. Subsequently, 21 studies progressed to full-text review, after which 11 were deemed eligible for inclusion in the final synthesis (see Figure 1 below for the PRISMA flow diagram illustrating the screening and study selection process). To ensure methodological rigor, two reviewers independently assessed all full-text articles against the stipulated criteria and resolved discrepancies through discussion and consultation within the review team to reach consensus. Ultimately, a total of 10 studies met the final inclusion criteria and were included in the systematic review.

**Figure 1:**
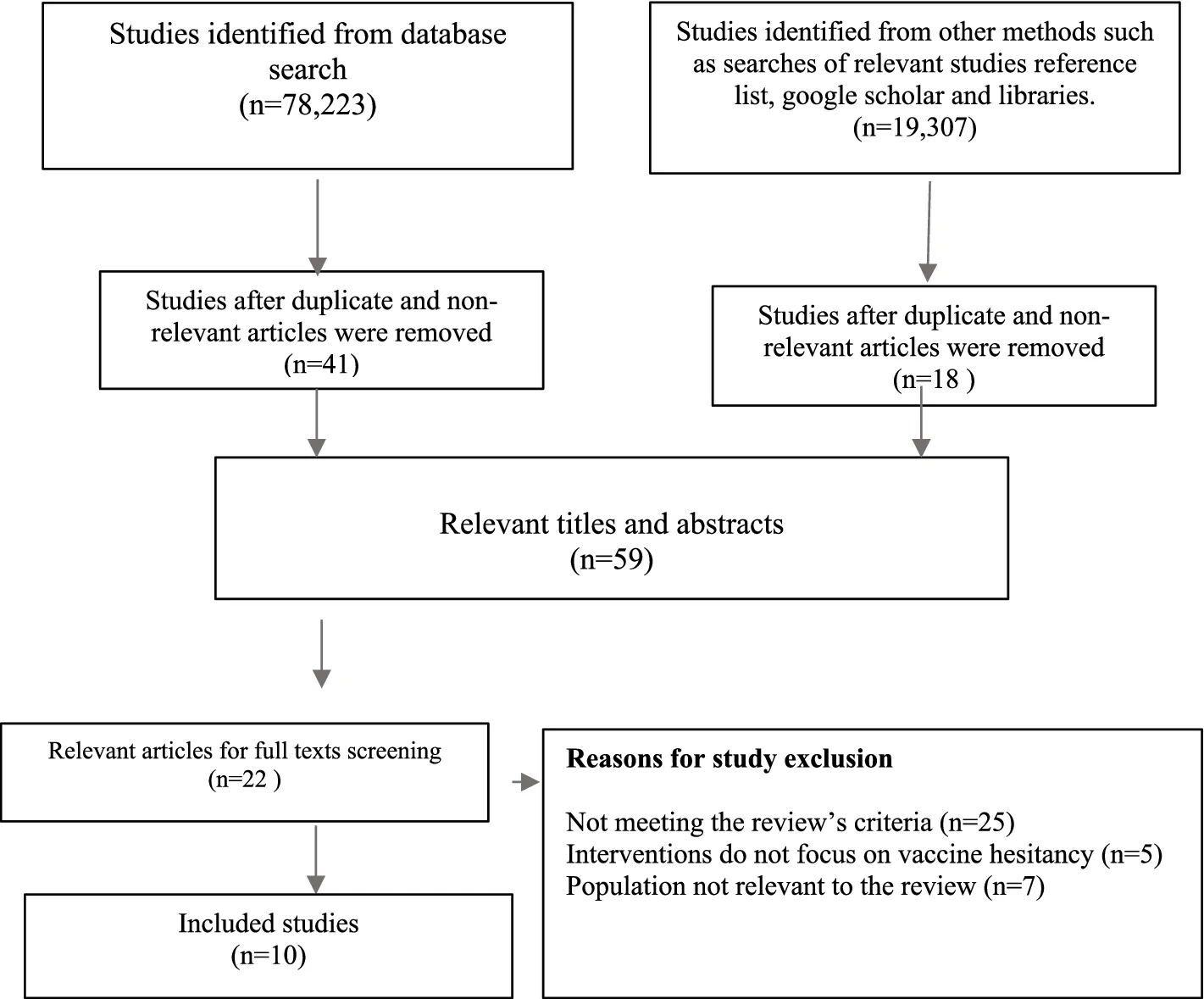
PRISMA flow diagram showing trajectory of the literature review process.

### Assessment of risk of bias

The methodological quality of the included studies was rigorously appraised using established and widely recognized critical appraisal tools tailored to the specific study designs. Qualitative studies that met the inclusion criteria were assessed using the Joanna Briggs Institute (JBI) Critical Appraisal Checklist for Qualitative Research, a comprehensive 10-item instrument designed to evaluate methodological congruity and interpretive credibility. This tool examines key domains, including alignment between the research methodology and the interpretation of findings, consistency between the stated philosophical perspective and the methodological approach, and other criteria. Each item was systematically rated as “Yes,” “No,” “Unclear,” or “Not applicable,” allowing for a structured and transparent assessment of study quality. For mixed-methods studies, the Critical Appraisal Skills Programme (43) tool was employed, consisting of a 10-question framework that facilitates the systematic evaluation of methodological rigor, data collection processes, and the appropriateness of qualitative and quantitative approaches. The CASP tool also allows reviewers to assign responses such as “Yes,” “No,” or “Cannot tell,” while providing space to justify these judgments, thereby enhancing the transparency and reflexivity of the appraisal process. Notably, CASP was also utilized to assess the potential risk of bias within the qualitative components of mixed-methods studies. For randomized controlled trials, the JBI Critical Appraisal Checklist for Randomized Controlled Trials, comprising 14 items, was applied to evaluate internal validity and methodological robustness. In addition, quantitative studies were appraised using the quality assessment checklist developed by Symonds and Tang (41), ensuring a comprehensive evaluation of bias and study reliability. Overall, the studies included in this review demonstrated a low risk of bias, indicating a strong evidence base underpinning the synthesis (see Appendix Table 2 for detailed risk-of-bias appraisal outcomes across all included studies).

#### Data synthesis: thematic coding, critical appraisal and synthesis

The key findings from the included studies were systematically synthesized using a rigorous thematic approach informed by Braun and Clarke (15) and guided by Torraco’s (16) integrative conceptual framework for literature synthesis, directly addressing the review’s research question.

A rigorous and transparent thematic synthesis was undertaken to identify and integrate evidence on the factors and barriers influencing childhood vaccination hesitancy among parents from ethnic minority backgrounds in the United Kingdom, alongside interventions designed to improve vaccination uptake. Following repeated reading and familiarization with the extracted findings, relevant text from each included study was coded line-by-line using an inductive approach, while remaining sensitive to concepts identified in the review questions. Codes captured both recurring and divergent perspectives on vaccination beliefs, trust, cultural and religious influences, misinformation, previous healthcare experiences, accessibility, communication, structural barriers, and intervention acceptability. Similar codes were compared, refined and grouped into descriptive categories, which were subsequently developed into higher-order analytical themes that explained how individual, interpersonal, cultural, community and healthcare-system factors shaped vaccination hesitancy and uptake. The lead reviewer coded systematically, and a second reviewer independently reviewed the coding, comparing emerging codes, categories, and themes against the original study findings to ensure they accurately represented participants’ accounts and intervention evidence. The team resolved coding or thematic interpretation differences through discussion and re-examination of the source data, and referred unresolved disagreements to a third reviewer to reach consensus.

Methodological quality was also critically appraised independently by two reviewers using the appropriate appraisal tool for each study design; reviewers compared assessments item-by-item, documented the rationale for judgements and resolved discrepancies through consensus, with a third reviewer available for adjudication where agreement could not be reached. Importantly, appraisal findings were not used simply to exclude studies or generate an overall quality score, but were integrated into the interpretation of the synthesis. Greater interpretive weight was placed on themes consistently supported by methodologically robust studies, while findings derived predominantly from studies with important methodological limitations were interpreted more cautiously. The appraisal therefore informed judgements about the strength, consistency and credibility of the evidence underpinning each theme, enabled contradictory findings to be examined in relation to study quality and context, and helped distinguish well-supported barriers and intervention approaches from findings requiring further investigation. Together, these procedures strengthened the transparency, reflexivity and methodological rigor of the synthesis and ensured that conclusions about vaccination hesitancy and interventions to improve childhood vaccination uptake among UK ethnic minority communities reflected both the content and the quality of the available evidence.

To strengthen analytical depth, a synthesis matrix was developed and used as a structured tool to critically evaluate each study’s methodological strengths, limitations, and identified gaps, ensuring a transparent and comprehensive comparison across studies. The synthesis process was conducted through a clearly defined four-stage procedure: (i) the systematic implementation of the literature search to identify relevant studies; (ii) the extraction and identification of key elements, concepts, and core ideas from each study; (iii) the organization and categorization of these elements into meaningful clusters to support pattern recognition; and (iv) the integration and synthesis of the data to generate new insights and, where appropriate, inform the development of future research or intervention directions. To further enhance analytical rigor at the final stage, an ‘index card method’ was employed, allowing for the effective sorting, comparison, and thematic grouping of key concepts emerging from earlier stages, thereby ensuring a robust, traceable, and conceptually rich synthesis of the included evidence.

#### Findings

Based on the thematic synthesis of relevant studies, the following themes emerged, including (i) Skepticism about vaccines, (ii) Distrust in health professionals and policy, (iii) Poor understanding and limited information about vaccines, (iv) Social network and family influence and (v) communication-based intervention. Below is a table summarizing each theme and corresponding studies (Table 2).

**Table 2:** Showing themes and corresponding studies.

S/n	Themes	Studies
i	Skepticism about vaccines	(6–8, 17–19)
ii	Distrust in health professionals and policy	(6, 7, 18–20)
iii	Poor understanding and limited information about vaccines	(6, 7, 10, 17, 19)
iv	Social network and family influence	(7, 17–21)
v	Communication-based intervention	(21, 22)

#### Skepticism about vaccines

Some studies report skepticism about vaccines and their link to other ailments such as autism (6–8, 17–19). For example, the study by Brown et al. (6) comprises 24 mothers who postponed or declined the first set of Measles, Mumps and Rubella (MMR) vaccines due to their perceived link or second-hand experience of an MMR-autism link and their lived experience of mild vaccine-related adverse effects. Another study comprising 23 Somali parents with children under 5 years old having low-level preschool immunization in Birmingham found that there was significant suspicion about the MMR vaccine, and this was largely due to anxiety about the perceived link with autism (19). One study comprising 5 parents, some identifying as Ghanaian and British black, identified skepticism about the pain that emanated from vaccination as a driving factor influencing the decision to immunize one’s child (8). Another study by Marlow, Wardle and Waller (17), comprising 20 Asian and black mothers in Britain, found that concerns around the side effects of vaccines were also reported by mothers as it relates to the implications of vaccines.

#### Distrust in health professionals and policy

A dominant theme across the synthesized studies was the perceived influence (20), including a lack of confidence in health professionals, political leaders and policy involved in the provision of the vaccines (6, 7, 18, 19). Brown et al. (6) found that MMR rejectors often shared experiences that undermined their trust in health professionals, thereby rationalizing their decision not to engage in MMR vaccination. Regarding trust, another study by Petts and Niemeyer (7) highlights how such trust was perceived to extend to political leaders, which was considered an important issue. A key finding was that all groups expressed concern about the Prime Minister’s refusal to say whether his son had received MMR and his failure to raise suspicion that something was wrong (7). As the leader of the Government, most participants felt he was supposed to inspire; that if it’s good enough for Leo Blair, then it’s good enough for their kids (7).

#### Poor understanding and limited information about vaccines

Several studies reported a poor understanding of vaccines (7, 17), including inadequate information to inform their decision to vaccinate (6, 10, 19). As Brown et al. (6) found, parents expressed frustration at the lack of accurate, unbiased information. Some official sources were perceived as wilfully misleading, whilst unofficial sources were deemed well-intentioned but unreliable. Another study by Condon (10), involving 21 Somali, Pakistani and African-Caribbean women, found that Afro-Caribbean and Somali women wanted more detailed information from health professionals, particularly written information about immunization that is inclusive of Black people and available in translated versions. In addition, limited information manifested as poor awareness. For example, one study by Marlow, Wardle and Waller (17), which explored attitudes to HPV vaccination among Asian and black mothers living in Britain, found that most mothers were unaware of the vaccine or its importance.

#### Social network and family influence

Some studies reported the role of social networks, including friends (7, 18, 19) and family influence, including husbands, or even grandparents, in influencing decisions regarding vaccine uptake (17, 20, 21). The study by Mixer et al. (20), comprising samples of mothers from Afro-Caribbean, Asian, and other ethnicities, found that grandparents influence decisions about immunization. Concerning the role of friends, a study conducted by Sythes and Bedford (18) found that 10 African Caribbean parents included 10 participants who were African Caribbean parents and found that friends influence parents on issues around vaccines, which often trigger hesitancy.

#### Communication-based intervention

A few studies reported the use of primary care-focused interventions, including communication-based interventions and a call-and-recall system (21, 22). Cockman et al. (22) conducted an observational time-series study to examine the effects of financial support for the development of geographically based networks and care package commissioning. It also examined the effects of the innovative use of information technology to enable active follow-up of defaulters and to increase knowledge about the demographics of the children most difficult to reach (22). The study found that developing networks of practices facilitated primary care clinicians and other stakeholders’ collaborative working, peer review of achievements, and healthy competition (22). Uptake improved for all childhood vaccinations and reached herd immunity levels for most. Uptake of the first dose of MMR vaccine before age 2 years rose from 80% in September 2009 to 94% by March 2011 (22). Drawing on a call-and-recall intervention examined qualitatively, Ali, Bell and Mounier-Jack (21) found that it was helpful in driving positive vaccine uptake among the Bangladeshi underserved ethnic minority population in the East London borough of Tower Hamlets.

## Discussions, implications, strengths and limitations

The review examined the factors and barriers influencing hesitancy among parents from ethnic minority backgrounds toward childhood vaccination, as well as interventions to encourage uptake in the United Kingdom. Informed by a systematic review and thematic synthesis, the findings yielded the following main themes: (i) Skepticism about vaccines, (ii) Distrust in health professionals and policy, (iii) Poor understanding and limited information about vaccines, (iv) Social network and family influence and (v) Communication-based intervention.

A major finding from the synthesized studies was that the persistently low childhood vaccine uptake among parents and caregivers from ethnic minority communities in the United Kingdom is underpinned by deeply rooted skepticism concerning vaccine safety, particularly the perceived link between vaccines and conditions such as autism (6–8, 17–19). These concerns appear to echo longstanding global and UK-specific evidence demonstrating how discredited, yet enduring narratives continue to shape parental risk perceptions (23, 24). A plausible explanation within ethnic minority communities is that culturally embedded health beliefs, intergenerational knowledge, and historical experiences of marginalization could further amplify such skepticism, fostering alternative explanatory views that prioritize natural immunity or traditional remedies over biomedical interventions. This aligns with wider literature suggesting that vaccine hesitancy is often tied to competing epistemologies and culturally situated understandings of health and illness (25, 42). Consequently, interventions that fail to engage with these socio-cultural dimensions risk reinforcing resistance rather than improving uptake.

Trust in healthcare systems, professionals, and government institutions emerged as a critical and cross-cutting determinant of vaccine acceptance among ethnic minority populations (6, 7, 18–20). Consistent with existing UK and international research, diminished institutional trust—often shaped by experiences of discrimination, perceived neglect, and lack of representation—undermines confidence in vaccination programs (26, 27). The findings illustrate how both interpersonal interactions with healthcare providers and broader political behaviors influence trust, with perceived opacity or inconsistency in public health messaging reinforcing suspicion. This reflects Giddens’ (28) conceptualization of *“abstract systems,”* where trust in expert systems is essential but fragile, especially when individuals feel excluded from decision-making. For ethnic minority communities, mistrust is not merely attitudinal but structurally produced, highlighting the need for culturally competent care, community engagement, and transparent communication strategies that actively rebuild credibility and legitimacy.

The synthesis further identifies significant informational barriers, with many ethnic minority parents reporting limited access to clear, culturally relevant, and linguistically appropriate vaccine information (6, 7, 10, 17, 19). This is consistent with broader evidence linking health literacy and communication inequalities to disparities in vaccine uptake (29, 30). Participants’ perceptions of official information as biased or insufficient underscore a critical gap in public health communication, particularly where messaging fails to reflect the lived realities and informational needs of diverse communities. Moreover, low awareness of certain vaccines—such as HPV—suggests systemic shortcomings in outreach and education strategies. These findings reinforce calls for co-produced, culturally tailored interventions that involve community leaders and utilize trusted communication channels to enhance both understanding and engagement (27, 31).

Furthermore, the influence of social networks, friends and family structures demonstrates that vaccine decision-making within ethnic minority communities is inherently relational and collectively negotiated (7, 17–21). In line with social ecological and network theories, health behaviors are shaped not only by individual beliefs but also by interpersonal dynamics and community norms (32, 33). The prominent role of family members—particularly elders and partners—reflects the importance of intergenerational authority and cultural continuity in shaping health decisions. This is supported by evidence that vaccine attitudes, including hesitancy and acceptance, are socially transmitted within networks, often reinforcing shared beliefs and experiences (24, 25). As such, interventions targeting individual parents in isolation are unlikely to be effective; instead, strategies that engage families, community influencers, and peer networks are essential to shifting norms and improving vaccine uptake within ethnic minority populations in the UK.

Finally, primary care–focused interventions, particularly communication strategies and call–recall systems, are consistently shown to improve childhood vaccination uptake by strengthening both service delivery and parental engagement. The findings align with wider evidence demonstrating that reminder–recall interventions and provider-led communication significantly increase immunization rates through improved access, trust, and continuity of care (34, 35). Furthermore, the use of practice networks, financial incentives, and data-driven follow-up reflects broader literature emphasizing the importance of system-level integration and collaborative working in reducing missed vaccinations and addressing inequities (36). However, a key limitation of this evidence base is the relatively narrow representation of diverse ethnic groups in intervention studies, which limits the transferability of findings to multicultural contexts. This lack of inclusive, ethnicity-focused interventions limits the ability to conduct a robust meta-synthesis and hinders a deeper understanding of culturally specific barriers and facilitators to vaccine uptake, particularly among underrepresented and marginalized groups.

## Limitations and strengths

This systematic review has several limitations that should be considered when interpreting the findings. First, the included studies were predominantly qualitative, with relatively small, context-specific samples and few intervention studies, which may limit the generalizability of the findings across the diverse ethnic minority populations in the United Kingdom. Second, the heterogeneity of the included studies—in terms of population groups, vaccines examined, and methodological approaches—may constrain comparability and synthesis, limiting the ability to draw uniform conclusions. Third, reliance on self-reported data in primary studies introduces the risk of recall and social desirability bias, potentially shaping how participants articulated their views on vaccination. Finally, this review may have been limited by language restrictions, as it included only English-language studies, potentially excluding relevant studies, particularly those capturing underrepresented or marginalized voices. Collectively, these limitations suggest that while the review provides important insights into vaccine hesitancy among ethnic minority communities, the findings should be interpreted with caution and situated within a broader evidence base.

Despite this limitation, a key strength of this systematic review is its rigorous application of thematic synthesis to generate a nuanced, higher-order understanding of barriers to childhood vaccine uptake among ethnic minority populations in the United Kingdom. By systematically integrating qualitative evidence, the review moves beyond fragmented insights to offer a coherent and conceptually rich account of parental vaccine hesitancy as a multidimensional phenomenon shaped by intersecting factors, including skepticism about vaccine safety, mistrust in healthcare and political systems, informational inequities, and the influence of social and familial networks. Importantly, the review advances the literature by positioning these barriers within broader socio-cultural and structural contexts, rather than framing hesitancy as an individual knowledge deficit. Its original contribution is particularly evident in foregrounding the role of non-clinical psychosocial interventions—such as community engagement, culturally tailored communication, and family-centered approaches—as critical mechanisms for addressing hesitancy in contextually relevant, socially embedded ways. By centering the lived experiences of ethnic minority communities and identifying actionable, culturally responsive strategies, the review provides a strong empirical foundation and a practical framework to inform equitable public health interventions aimed at improving childhood vaccination uptake in the UK.

## Policy implications

The findings of this review have significant implications for public health policy aimed at improving childhood vaccine uptake among ethnic minority population in the United Kingdom. First, addressing vaccine skepticism requires a shift from deficit-based models of health education towards culturally grounded, trust-sensitive approaches. Policymakers should prioritize the co-production of interventions with ethnic minority communities to directly counter misinformation—particularly enduring myths such as vaccine–autism links—through credible, community-endorsed narratives. This includes investing in culturally competent health promotion campaigns that integrate lived experience, acknowledge historical concerns, and engage trusted community leaders, faith organizations, and peer advocates as key messengers.

Second, rebuilding trust in healthcare systems and government institutions is encouraged to be treated as a central policy priority rather than an adjunct to communication strategies. Policies should mandate and resource culturally competent care, anti-discriminatory practices, and inclusive service delivery within primary healthcare settings. Transparency and consistency in public health messaging are essential, along with visible leadership accountability, to reinforce confidence in vaccination programs. Embedding community partnership models where ethnic minority communities actively shape, deliver, and evaluate vaccination services, can help repair fractured trust and strengthen legitimacy.

Third, the identified informational barriers highlight the need for a more equitable and accessible health communication infrastructure. Policy initiatives should ensure that vaccine information is not only accurate but also culturally relevant, linguistically appropriate, and tailored to varying levels of health literacy. This includes developing multilingual materials, visual resources, and digital platforms that reflect the diversity of ethnic minority populations. Crucially, information dissemination strategies should move beyond passive provision to active engagement, such as community workshops, outreach programs, and culturally adapted education sessions delivered in trusted community spaces.

Finally, recognizing the significant influence of social networks and family structures, policy interventions should adopt a whole-community and family-centered approach to vaccine promotion. Strategies should target not only parents but also influential figures within family and community systems, including partners, grandparents, and community elders. Supporting peer-led interventions, community champions, and social network-based outreach can facilitate the diffusion of accurate information and positive vaccine norms. Collectively, these recommendations underscore the need for integrated, culturally responsive, and socially embedded policy frameworks that address the complex interplay of trust, knowledge, cultural beliefs, and relational dynamics shaping vaccine hesitancy among ethnic minority populations in the UK.

## Conclusion

In conclusion, this systematic review provides a robust and contextually grounded understanding of the multifaceted barriers underpinning poor childhood vaccine uptake among ethnic minority populations in the United Kingdom. By synthesizing evidence through a thematic approach, the review demonstrates that parental vaccine hesitancy is shaped by an interplay of entrenched skepticism, diminished trust in health and political institutions, persistent informational inequities, and the powerful influence of social and familial networks. Crucially, these findings challenge reductive, individual-level explanations of hesitancy and instead foreground the socio-cultural and structural determinants that sustain disparities in vaccine uptake. The review makes a significant contribution by highlighting the importance of non-clinical, psychosocial, and culturally responsive interventions, particularly those that are co-produced, community-led, and family-centered as essential strategies for improving engagement and rebuilding trust. Overall, addressing vaccine hesitancy within ethnic minority communities requires a shift towards inclusive, equity-driven public health approaches that recognize and respond to the lived realities, belief systems, and relational dynamics that shape health decision-making.

## Data Availability

The review did not generate any original data and all data relied on are cited and available in the public domain. Further inquiries can be directed to the corresponding author/s.
