# Supplementary material for: Factors influencing childhood vaccine hesitancy and interventions for parents and caregivers from ethnic minority backgrounds in the United Kingdom to encourage uptake: a systematic review
Source: Front Public Health. 2026 Oct 12;14:1929472. doi: 10.3389/fpubh.2026.1929472 (PMC13696398; doi:10.3389/fpubh.2026.1929472)
Supplement: Supplementary file 1 [file Table_1.docx]

**Appendix 1: Study Characteristics**

| Author(s) | Aims | Population | Methods | Findings | Strengths | Limitation(s) |
| --- | --- | --- | --- | --- | --- | --- |
| Ali, Bell & Mounier-Jack, (2025) | The study explored key enablers for early childhood vaccination uptake among the Bangladeshi community in East London, UK. | 23 Bangladeshi parents 11 primary healthcare professionals (HCPs) and 5 community service providers (CSPs) involved in delivering childhood vaccination services. | A qualitative study using semi-structured interviews | This study found that multi-level trust in a vaccination programme could propel positive vaccine uptake in ethnic minority population. It also found that person-centred delivery of vaccination services, including cultural sensitivity, alongside leveraging community dynamics and trusted social networks, are imperative to address the informational, linguistic and cultural needs that facilitate vaccine uptake within the Bangladeshi community. | Focus on an underserved ethnic minority group. | Small sample size and only focused on one ethnic minority group which could limit generalisability to other ethnic minority groups. |
| Browne et al., (2012) | The study explored parents’ MMR decision-making. | 24 mothers planning to accept, postpone or decline the first MMR dose (MMR1) for their 11–36 month-old children, described their decision-making. | Qualitative methods incorporating semi-structured interviews. | The study found that parents opting for single vaccines expressed the sense that immune overload is not a consideration, but that not all three components of MMR are warranted by disease severity; and MMR1 rejectors openly criticised other parents’ MMR decisions and decision-making. | Involved data collection through semi-structured interviews. | Small sample size. |
| Cockman et al., (2011) | The purpose of the study was to increase MMR vaccination rates to 95% to prevent further outbreaks of measles in Tower Hamlet  borough. | Ethnic minorities. | Observational time series study | The study found that networks of practices development facilitated collaborative working among primary care clinicians and other  stakeholders; peer review of achievements; and an element of healthy competition. Uptake improved for all childhood vaccinations, and to herd  immunity levels for most. Uptake of the first MMR vaccine before age 2 years rose from 80% in September 2009 to 94% in March 2011. | Focused on ethnic minority. | Focused on only Tower Hamet Borough which might have implications for generalisation. |
| Condon (2002) | To explore the attitudes of parents from ethnic minority background as it relates to preschool  immunisations, particularly first MMR  immunisation. | Mothers of children aged 16  months to 3 years  of Afro-Caribbean (5), Pakistani (11), and Somali (5)  ethnicity. | Semi-structured  interviews  and focus  groups. | The study found that three ethnic groups had different exposure to media publicity about the risks of MMR, largely linked to command of English. Pakistani women believed that health professionals gave them the best advice about childhood immunisation, but Afro-Caribbean and Somali women expressed a desire for more information from health professionals, particularly written information about immunisation which is inclusive of Black people and available in translation. | Focused on ethnic minority. | Small sample size. |
| Hill & Cox, (2013) | To determine and examine the factors  influencing parental immunisation decision making. | Parents of  children who have  received the MMR  immunisation. 1; Ghanaian, 1;  Turkish, 1; British  Black  Language spoken  was English | Semi-structured  interviews | The study found that the practice nurse appears to constitute a credible source of information for parents seeking informed decision making. | Focused on ethnic minority group. | Small sample size. |
| Marlow, Wardle and Waller, (2009) | To explore Black and Asian mothers’ attitudes to HPV immunisation in Britain. | 20 Black and Asian  mothers living in  Britain. 6; Black Caribbean, 4; Black African, 3; Asian  Indian, 3; Asian  Pakistani, 1; Asian  Bangladeshi, 3; Asian  other | Interviews | The study found that visits to family abroad meant additional experience with vaccinations.  There were concerns about how vaccine effects could vary by ethnicity as a result of physical differences (e.g. sickle-cell anaemia), and mothers wanted to know whether the HPV vaccine had been tested fully in their ethnic group. | Focused on ethnic minority group. | Small sample size. |
| Mixer, Jamrozik & Newsom, (2007) | The study explored ethnic  differences in  attitudes, knowledge,  and behaviour related  to immunisation. | 24 Mothers from ethnic  Backgrounds, including 12 Afro-Caribbean, and 12  Asian. | Mixed methods | The qualitative data suggest clear differences between the ethnic groups with respect to awareness of the controversy surrounding MMR vaccination (related to use of English-language media) and influence of grandparents and health  professionals in decisions about immunisation. A multiple logistic regression model showed that although  coverage of MMR vaccination increased with increasing socioeconomic status, there was no evidence of a  statistically significant interaction between socioeconomic status and ethnicity. | Focused on ethnic minority group. | Small sample size. |
| Petts & Niemeyer (2004) | The study examined the information strategies that parents adopt to make sense of health risk issues | Parents who had children with various MMR  Immunisation status with at least 28 Asian Muslim mothers. | Focus group | The study found the importance of social networks in reinforcing parental understanding and beliefs. While the media are identified as important sources of information, there is no evidence to suggest that parents passively receive and act upon such risk  messages. | Focused on ethnic minority group. | Small sample size. |
| Sythes & Bedford (2022) | The study sought to examine the interaction between gender, identity and non-vaccination, as well as the non-vaccination journey for parents in the United Kingdom. | 10 mothers comprising African Caribbean | Semi-structured  interviews | The study found that within shared parenting priority, identities varied considerably.  Most mothers strongly rejected the label or identity of ‘anti-vaxxer’, preferring  alternative terms with less negative social connotations. The study also found that the decision not to vaccinate was predominantly made by mothers. | Include ethnic minority group. | Small sample size. |
| Tomlinson & Redwood, (2013) | The aim of the study was to explore the Somali women resident in the UK health  Beliefs. | 23 Somali women  resident in the  UK with one  child <5 years  of age | Semi-structured  interviews | The study found that attitudes towards preschool immunisations were quite positive. However, participants expressed significant suspicion about the MMR vaccine, predominantly due to anxiety about a link with autism. Beliefs were affected by social and cultural interpretations of Islam and practices associated with them. | Focused on ethnic minority group. | Small sample size and focus on only one ethnic group thus, limiting its generalisability. |

**Appendix 2: The Joanna Briggs Institute (JBI) Critical Appraisal Checklist for Qualitative Research**

| Author(s) | 1. Is there congruity between the stated philosophical perspective and the research methodology?  Yes/No/Unclear/ Not applicable | 2. Is there congruity between the research methodology and the research question or objectives? Yes/No/Unclear/ Not applicable | 3. Is there congruity between the research methodology and the methods used to collect data? Yes/No/Unclear/ Not applicable | 4. Is there congruity between the research methodology and the representation and analysis of data? Yes/No/Unclear/ Not applicable | 5. Is there congruity between the research methodology and the interpretation of results? Yes/No/Unclear/ Not applicable | 6. Is there a statement locating the researcher culturally or theoretically? Yes/No/Unclear/ Not applicable | 7. Is the influence of the researcher on the research, and vice- versa, addressed? Yes/No/Unclear/ Not applicable | 8. Are participants, and their voices, adequately represented? Yes/No/Unclear/ Not applicable | 9. Is the research ethical according to current criteria or, for recent studies, and is there evidence of ethical approval by an appropriate body? Yes/No/Unclear/ Not applicable | 10. Do the conclusions drawn in the research report flow from the analysis, or interpretation, of the data? Yes/No/Unclear/ Not applicable | 11. Overall appraisal: include, exclude, seek further info | 12.Comments including reasons for exclusion |
| --- | --- | --- | --- | --- | --- | --- | --- | --- | --- | --- | --- | --- |
| Ali, Bell & Mounier-Jack, (2025) | Yes | Yes | Yes | Yes | Yes | No | No | Yes | Yes | Yes | Include | The research focussed on the target population and met the review inclusion criteria. |
| Browne et al., (2012) | Yes | Yes | Yes | Yes | Yes | No | Unclear | Yes | Yes | Yes | Include | Met the review inclusion criteria. |
| Sythes & Bedford (2022) | Yes | Yes | Yes | Yes | Yes | No | No | Yes | Yes | Yes | Include | The research showed transparency in data reporting and also met the inclusion criteria. |
| Condon (2002) | Yes | Yes | Yes | Yes | Yes | Unclear | Unclear | Yes | Yes | Yes | Include | Met the review inclusion criteria. |
| Hill & Cox, (2013) | Yes | Yes | Yes | Yes | Yes | Unclear | No | Yes | Yes | Yes | Include | Met the review inclusion criteria. |
| Marlow, Wardle and Waller, (2009) | Yes | Yes | Yes | Yes | Yes | Yes | No | Yes | Yes | Yes | Include | Met the review inclusion criteria. |
| Petts & Niemeyer (2004) | Yes | Yes | Yes | Yes | Yes | No | Unclear | Yes | Yes | Yes | Include | Met the review inclusion criteria. |
| Sythes & Bedford (2022) | Yes | Yes | Yes | Yes | Yes | No | No | Yes | Yes | Yes | Include | Include |

**Appendix 3: Quality Appraisal Checklist for Quantitative, Qualitative, and Mixed Methods Studies (Symonds & Tang et al., 2024)**

| Author(s) | 1. Was the study setting (venues) and/or geographic location described in detail? | 2. Were the recruitment processes explained? | 3. Was the administration process explained? | 4. Did the researchers receive informed consent from human participants? | 5. Is there a clear statement about ethical practices? | 6. Was the study sample described in detail? | 7. Were the numbers of people whodid/did not consent to participate stated? | 8. Was the number of participants justified? | 9. Was the missingness of data mentioned? | 10. Was the number of participants at each stage/ wave specified? | 11. Did the authors report their missing data handling strategy? | 12. Was the reliability and validity of measurement methods mentioned? | 13.  For studies with repeated measures / longitudinal data |
| --- | --- | --- | --- | --- | --- | --- | --- | --- | --- | --- | --- | --- | --- |
| Mixer, Jamrozik & Newsom, (2007) | Yes | Yes | Yes | Yes | Yes | Yes | Yes | Yes | Unclear | Yes | Not clear | Yes | N/a |
| Cockman et al., (2011) | Yes based on the observational time series design | Yes | Yes | Unclear | Yes | Yes | N/A | Yes | Unclear | Yes | Unclear | Unclear | N/A |

**Appendix 6: PRISMA Checklist**

| **Section and Topic** | **Item #** | **Checklist item** | **Location where item is reported** |
| --- | --- | --- | --- |
| **TITLE** | | |  |
| Title | 1 | Identify the report as a systematic review. | Title and abstract |
| **ABSTRACT** | | |  |
| Abstract | 2 | See the PRISMA 2020 for Abstracts checklist. | Abstract |
| **INTRODUCTION** | | |  |
| Rationale | 3 | Describe the rationale for the review in the context of existing knowledge. | Introduction |
| Objectives | 4 | Provide an explicit statement of the objective(s) or question(s) the review addresses. | Introduction |
| **METHODS** | | |  |
| Eligibility criteria | 5 | Specify the inclusion and exclusion criteria for the review and how studies were grouped for the syntheses. | Methods |
| Information sources | 6 | Specify all databases, registers, websites, organisations, reference lists and other sources searched or consulted to identify studies. Specify the date when each source was last searched or consulted. | Methods |
| Search strategy | 7 | Present the full search strategies for all databases, registers and websites, including any filters and limits used. | Methods |
| Selection process | 8 | Specify the methods used to decide whether a study met the inclusion criteria of the review, including how many reviewers screened each record and each report retrieved, whether they worked independently, and if applicable, details of automation tools used in the process. | Methods |
| Data collection process | 9 | Specify the methods used to collect data from reports, including how many reviewers collected data from each report, whether they worked independently, any processes for obtaining or confirming data from study investigators, and if applicable, details of automation tools used in the process. | Methods |
| Data items | 10a | List and define all outcomes for which data were sought. Specify whether all results that were compatible with each outcome domain in each study were sought (e.g. for all measures, time points, analyses), and if not, the methods used to decide which results to collect. | Methods |
|  | 10b | List and define all other variables for which data were sought (e.g. participant and intervention characteristics, funding sources). Describe any assumptions made about any missing or unclear information. | Methods and Appendix |
| Study risk of bias assessment | 11 | Specify the methods used to assess risk of bias in the included studies, including details of the tool(s) used, how many reviewers assessed each study and whether they worked independently, and if applicable, details of automation tools used in the process. | Methods and Appendix |
| Effect measures | 12 | Specify for each outcome the effect measure(s) (e.g. risk ratio, mean difference) used in the synthesis or presentation of results. | Not applicable |
| Synthesis methods | 13a | Describe the processes used to decide which studies were eligible for each synthesis (e.g. tabulating the study intervention characteristics and comparing against the planned groups for each synthesis (item #5)). | Methods and appendix |
|  | 13b | Describe any methods required to prepare the data for presentation or synthesis, such as handling of missing summary statistics, or data conversions. | Not applicable |
|  | 13c | Describe any methods used to tabulate or visually display results of individual studies and syntheses. | Methods and appendix |
|  | 13d | Describe any methods used to synthesize results and provide a rationale for the choice(s). If meta-analysis was performed, describe the model(s), method(s) to identify the presence and extent of statistical heterogeneity, and software package(s) used. | Methods and appendix |
|  | 13e | Describe any methods used to explore possible causes of heterogeneity among study results (e.g. subgroup analysis, meta-regression). | Not applicable |
|  | 13f | Describe any sensitivity analyses conducted to assess robustness of the synthesized results. | Methods and appendix |
| Reporting bias assessment | 14 | Describe any methods used to assess risk of bias due to missing results in a synthesis (arising from reporting biases). | Methods and appendix |
| Certainty assessment | 15 | Describe any methods used to assess certainty (or confidence) in the body of evidence for an outcome. | Methods and appendix |
| **RESULTS** | | |  |
| Study selection | 16a | Describe the results of the search and selection process, from the number of records identified in the search to the number of studies included in the review, ideally using a flow diagram. | Methods and appendix |
|  | 16b | Cite studies that might appear to meet the inclusion criteria, but which were excluded, and explain why they were excluded. | Methods |
| Study characteristics | 17 | Cite each included study and present its characteristics. | Methods, findings and appendix |
| Risk of bias in studies | 18 | Present assessments of risk of bias for each included study. | Methods and appendix |
| Results of individual studies | 19 | For all outcomes, present, for each study: (a) summary statistics for each group (where appropriate) and (b) an effect estimate and its precision (e.g. confidence/credible interval), ideally using structured tables or plots. | Not applicable |
| Results of syntheses | 20a | For each synthesis, briefly summarise the characteristics and risk of bias among contributing studies. | Methods and appendix |
|  | 20b | Present results of all statistical syntheses conducted. If meta-analysis was done, present for each the summary estimate and its precision (e.g. confidence/credible interval) and measures of statistical heterogeneity. If comparing groups, describe the direction of the effect. | Findings |
|  | 20c | Present results of all investigations of possible causes of heterogeneity among study results. | Not applicable |
|  | 20d | Present results of all sensitivity analyses conducted to assess the robustness of the synthesized results. | Methods and appendix |
| Reporting biases | 21 | Present assessments of risk of bias due to missing results (arising from reporting biases) for each synthesis assessed. | Methods and appendix |
| Certainty of evidence | 22 | Present assessments of certainty (or confidence) in the body of evidence for each outcome assessed. | Methods and appendix |
| **DISCUSSION** | | |  |
| Discussion | 23a | Provide a general interpretation of the results in the context of other evidence. | Findings and discussion |
|  | 23b | Discuss any limitations of the evidence included in the review. | Discussion |
|  | 23c | Discuss any limitations of the review processes used. | Discussion |
|  | 23d | Discuss implications of the results for practice, policy, and future research. | Discussion |
| **OTHER INFORMATION** | | |  |
| Registration and protocol | 24a | Provide registration information for the review, including register name and registration number, or state that the review was not registered. | Abstract and methods |
|  | 24b | Indicate where the review protocol can be accessed, or state that a protocol was not prepared. | Abstract and methods |
|  | 24c | Describe and explain any amendments to information provided at registration or in the protocol. | Not applicable |
| Support | 25 | Describe sources of financial or non-financial support for the review, and the role of the funders or sponsors in the review. | Not applicable |
| Competing interests | 26 | Declare any competing interests of review authors. | Not applicable |
| Availability of data, code and other materials | 27 | Report which of the following are publicly available and where they can be found: template data collection forms; data extracted from included studies; data used for all analyses; analytic code; any other materials used in the review. | Methods and discussion |
